# Rayleigh scattering in few-mode optical fibers

**DOI:** 10.1038/srep35844

**Published:** 2016-10-24

**Authors:** Zhen Wang, Hao Wu, Xiaolong Hu, Ningbo Zhao, Qi Mo, Guifang Li

**Affiliations:** 1 School of Precision Instrument and Optoelectronic Engineering, Tianjin University, Tianjin 300072, China; 2 Key Laboratory of Optoelectronic Information Science and Technology, Ministry of Education, Tianjin 300072, China; 3 Wuhan Research Institute of Posts and Telecommunications, Wuhan 430074, China; 4 CREOL, The College of Optics & Photonics, University of Central Florida, Orlando, FL 32816, USA

## Abstract

The extremely low loss of silica fibers has enabled the telecommunication revolution, but single-mode fiber-optic communication systems have been driven to their capacity limits. As a means to overcome this capacity crunch, space-division multiplexing (SDM) using few-mode fibers (FMF) has been proposed and demonstrated. In single-mode optical fibers, Rayleigh scattering serves as the dominant mechanism for optical loss. However, to date, the role of Rayleigh scattering in FMFs remains elusive. Here we establish and experimentally validate a general model for Rayleigh scattering in FMFs. Rayleigh backscattering not only sets the intrinsic loss limit for FMFs but also provides the theoretical foundation for few-mode optical time-domain reflectometry, which can be used to probe perturbation-induced mode-coupling dynamics in FMFs. We also show that forward inter-modal Rayleigh scattering ultimately sets a fundamental limit on inter-modal-crosstalk for FMFs. Therefore, this work not only has implications specifically for SDM systems but also broadly for few-mode fiber optics and its applications in amplifiers, lasers, and sensors in which inter-modal crosstalk imposes a fundamental performance limitation.

The discovery by Kao that the optical loss of silica fiber could be reduced to below 20 dB/km^1^ has spurred the growth of fiber optics and its applications in optical communication^2^. Today, optical fibers have become the backbone that supports the internet. Because of the exponentially-increasing bandwidth demand, optical-communication systems based on single-mode fibers have been driven to their capacity limits. Space-division multiplexing (SDM)^3^,^4^,^5^,^6^ including mode-division multiplexing (MDM)^7^ and core multiplexing, has been proposed as the next-generation solution to further increase the transmission capacity^8^,^9^,^10^,^11^,^12^,^13^,^14^,^15^,^16^. Ideally, an *N*-mode optical fiber could permit *N* channels to transmit independent data and therefore increasing the transmission capacity by a factor of *N*, in comparison with a single-mode fiber. Rayleigh scattering is an inherent process that will affect signal propagation in these channels. Additionally, crosstalk among these channels due to random mode-coupling^17^,^18^,^19^ induced either by perturbation or by fiber imperfections exists. Because inter-modal crosstalk renders these channels interdependent^17^,^20^, multiple-input-multiple-output^18^ (MIMO) digital-signal processing (DSP) is needed at the receiver for de-multiplexing the signals. Because the MIMO DSP complexity scales as *N*^2^, optical fibers with a few modes, or, so-called few-mode fibers (FMFs), have been deployed for SDM systems.

Rayleigh scattering has been studied in single- and multi-mode optical fibers. And the relevant theories are well established. In particular, with regard to the Rayleigh scattering theory developed for MMFs^21^, a mode-continuum model was adopted and an incoherent total power transfer formulism was established. However, mode-resolved Rayleigh scattering in few-mode fibers has not been systematically investigated. In this work, we establish a general model for Rayleigh scattering in FMFs and verify the model experimentally. The model permits analytical calculations of time-dependent, mode-resolved power of Rayleigh scattering, including the forward and backward, intra-modal and intermodal cases. This model not only can provide the theoretical foundation for optical time-domain reflectometry in few-mode fibers, but also be used to probe the random mode-coupling dynamics caused by perturbation. Furthermore, we found that even in absence of external perturbation and fiber imperfections, inter-modal crosstalk in FMF still exists due to the existence of forward, inter-modal Rayleigh scattering. Thus, it is the forward, inter-modal Rayleigh scattering that makes crosstalk an inerasable signature in FMFs and sets the crosstalk limit. Our illustration of the role of Rayleigh scattering in FMFs can guide future design and optimizations of hardware and algorithms of SDM transmission systems and networks. The general theoretical framework for Rayleigh scattering in FMFs, on the other hand, can be used to analyze the Rayleigh-scattering signals that are used for probing the mode-coupling dynamics in FMFs, and impact fiber lasers^22^, amplifiers^23^, and sensors^24^, in which FMFs are used and inter-modal crosstalk is therefore a fundamental limitation.

## Results

### Theory

We used a three-mode optical fiber, containing the LP_01_, LP_11a_, and LP_11b_ modes, as an example to study Rayleigh scattering. The theory we developed here, however, can be extended to an arbitrary number of modes. The experiment, in principle, can also be trivially extended with the availability of efficient multiplexers/demultiplexers. In this work we didn’t take polarizations into account and treated Rayleigh scattering as a scalar process.

We derived the equation for mode-resolved time-dependent power of Rayleigh back-scattering received at the front end of the fiber. To develop the theory, we assumed that an optical pulse, with a temporal width of *ΔT* and a constant power *P*_0_ was launched at *z* = 0 into mode *i* (*i* = 1, 2, 3) of the three-mode fiber. In the main text of the paper, as well as in the Supplementary Information (SI), we use subscripts 1, 2, and 3 to represent the LP_01_, LP_11a_, and LP_11b_ modes, respectively. The time-dependent power of Rayleigh back-scattering in mode *j*( *j* = 1, 2, 3) received at *z* = 0 can be calculated by


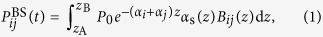


where 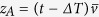; 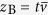; 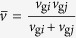; *v*_g*i*_ and *v*_g*j*_ are the group velocities of modes *i* and *j*, respectively; *α*_*i*_ and *α*_*j*_ are the optical attenuation coefficients of modes *i* and *j*, respectively; *α*_s_(*z*) is the ratio of the total scattered power at *z* to the incident power at *z*; *B*_*ij*_(*z*) is the overall capture fraction, quantifying the ratio of the scattered power into mode *j* to the total scattered power at *z*. Detailed derivation of Eq. (1) using space-time diagrams is presented in Section I of SI. If we further assume that *α*_s_(*z*) = *α*_s_, *B*_*ij*_(*z*) = *B*_*ij*_, and 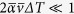 (see Section I of SI for justifications), where 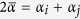, Eq. (1) can be approximated as


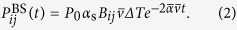


We note that Eq. (2) becomes identical to the equation for the single-mode case^25^ if we set *i* = *j* = 1, *v*_g*i*_ = *v*_g*j*_ = *v*_g_, and *α*_*i*_ =* α*_*j*_ = *α*.

In Eq. (2), the overall capture fraction, *B*_*ij*_, can be calculated by averaging the local capture fraction, *b*_*j*_(*R*, *ϕ*), over the near-field intensity distribution of the incident mode *i*:


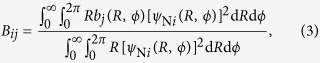


where


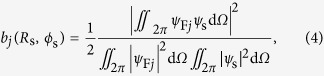


in which *ψ*_N*j*_ and *ψ*_F*j*_ represent the near and far fields (see Section II of SI) of mode *j*, respectively; *ψ*_s_ is the field radiated by a dipole (see Section III of SI). Rayleigh scattering at (*R*_s_, *ϕ*_s_) is modeled as the field of a dipole located at (*R*_s_, *ϕ*_s_). Following derivations detailed in Section IV of SI, we obtain *b*_*j*_(*j* = 1, 2, 3):


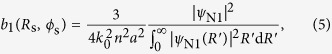



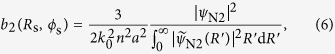


and


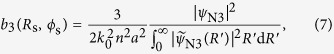


where *R*′ = *R*/*a*, *a* is the radius of the core of the optical fiber, 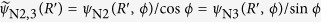. From Eqs (3), (5), (6) and (7), we obtain *B*_*ij*_:


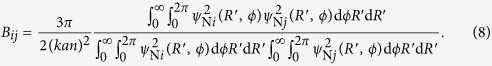


The parameters *B*_*ij*_ satisfy the following relations (see Section V of SI):


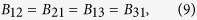


and


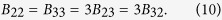


**Figure 1:**
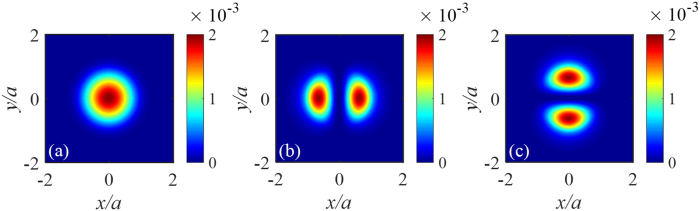
Local capture fraction of Rayleigh back-scattering in few-mode fibers. (**a**), (**b**) and (**c**) are for the excitations by *LP*_01_, *LP*_11*a*_, and *LP*_11*b*_ modes, respectively.

Figure 1 presents the local capture fraction, *b*_*j*_(* j* = 1, 2, 3). As expected [see Eqs (5), (6) and (7)], the distribution of *b*_*j*_ across the cross-section of the fiber closely mimics the intensity distribution of the near field of mode *j*. Indeed, we calculated *b*_*j*_ using Eq. (4) directly without approximations and found that the maximum relative difference was 6% in the core (see Section IV of SI). This comparison justifies the approximations we used.

**Figure 2:**
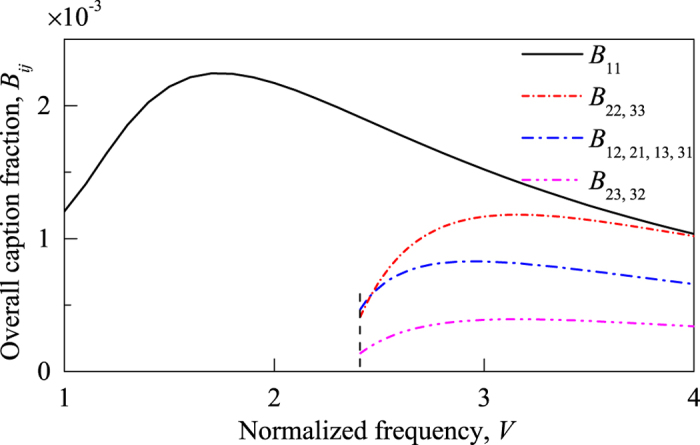
Overall capture fraction of Rayleigh back-scattering in few-mode fibers. The dashed vertical line corresponds to *V* = 2.405, the normalized cut-off frequency for the *LP*_11*a*_ and *LP*_11*b*_ modes.

Figure 2 presents the overall capture fraction, *B*_*ij*_, as a function of normalized frequency *V* ≡ 2*πa*NA/*λ*, where *a* is the core radius of the fiber, NA is the numerical aperture of the fiber, and *λ* is the wavelength in free space. The dashed line shows the normalized cut-off frequency for the LP_11a_ and LP_11b_ modes, at *V* = 2.405. Our experiment was performed at *V* = 3.49.

The degeneracy and the relative magnitudes of *B*_*ij*_ in Eqs (8) and (9) can be clearly seen in Fig. 2, and are understandable from the mode distribution and the associated symmetries. We note that our result of *B*_11_ is almost identical with the single-mode result reported in ref. 24 with discrepancies due to the differences of fiber parameters.

Before we further discuss the case of forward scattering and its impact on inter-modal crosstalk, we first verify the newly-developed model via OTDR measurement of the backscattering. This is because the theories for forward- and backward-scattering share significant similarities. After we place the backward-scattering case on a solid ground, we can develop the forward case by analogy.

### Experiment

**Figure 3:**
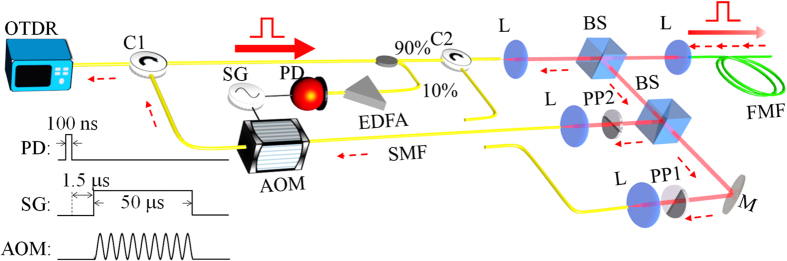
Schematic of the experimental setup used for measuring Rayleigh back-scattering in a three-mode optical fiber. A commercial OTDR was used for excitation and detection. Laser pulses from the OTDR, at the wavelength of 1550 nm, with 100-ns width and 37-mW peak power, were launched into a free-space mode-multiplexer/de-multiplexer and then into the three-mode optical fiber. The reflected light and Rayleigh back-scattering were demultiplexed, then went through an acousto-optic modulator (AOM), and finally went back into the OTDR through a circulator. The output of the photodetector triggers the signal generator; the output of the signal generator modulates the 55-MHz driving signal of the AOM, generating a 50-*μ*s window that the OTDR receives Rayleigh back-scattering. C: circulator; L: lens; BS: beam splitter; M: mirror; SMF: single-mode fiber; FMF: few-mode fiber; PP1: phase plate 1 for the *LP*_11*a*_ channel; PP2: phase plate 2 for the *LP*_11*b*_ channel; AOM: acousto-optic modulator; EDFA: erbium-doped fiber amplifier; PD: photodetector; SG: signal generator.

**Figure 4:**
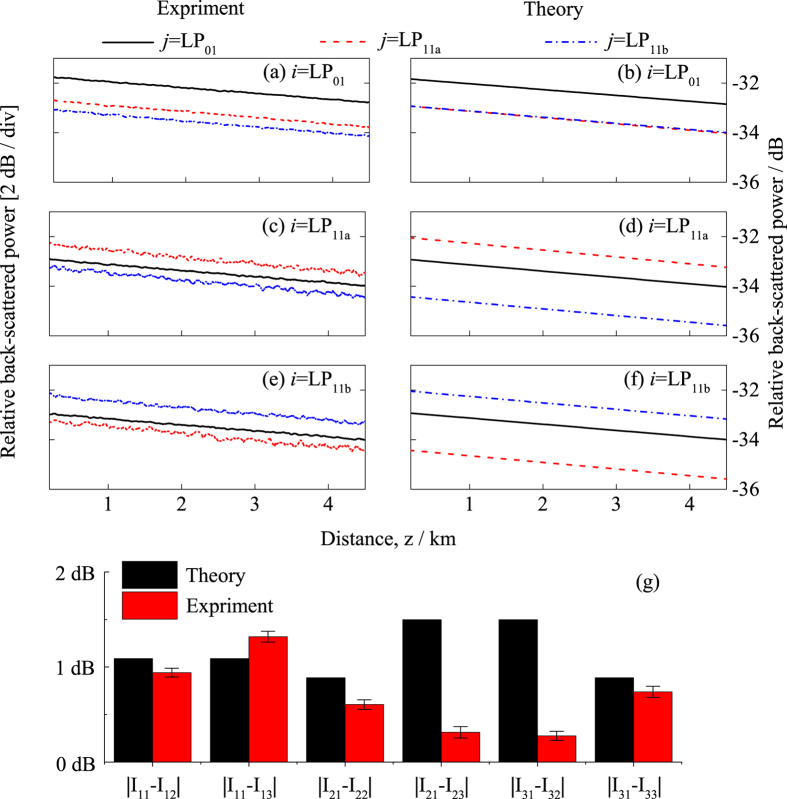
Optical-time-domain-reflectometer measurement of Rayleigh back-scattering in a three-mode optical fiber and the corresponding theoretical results. (**a**) experimental results with *LP*_01_ excitation; (**b**) theoretical results with *LP*_01_ excitation; (**c**) experimental results with *LP*_11*a*_ excitation; (**d**) theoretical results with *LP*_11*a*_ excitation; (**e**) experimental results with *LP*_11*b*_ excitation; (**f**) theoretical results with *LP*_11*b*_ excitation; (**g**) comparison of the experimental and theoretical intercepts relative to *I*_*i*1_.

We measured the time-dependent power of Rayleigh back-scattering using the experimental setup schematically shown in Fig. 3. The procedure of the measurement is detailed in the section of Methods. Figure 4 presents the experimental and theoretical results of time-dependent 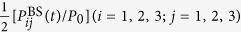:


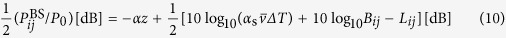


The experimental data were raw data obtained by OTDR measurement averaged over 30 seconds, taking into account the insertion and coupling losses, *L*_*ij*_. Section VI of SI presents the measurement of *L*_*ij*_ in detail. For calculating the theoretical curves, we used the values obtained by fitting the experimental data of 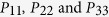 for *α*_*i*_ and *α*_*j*_; *α*_s_ ≈ −0.039/km as in ref. 26; *v*_g*i*_ ≈ *v*_g*j*_ ≈ 2.067 × 10^5^ km/s. To quantify the relative difference between theoretical and experimental results, we compare the intercepts at *z* = 0 of the OTDR curves, as relative to 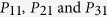, as shown in Fig. 4(g). The uncertainties in our measurement in Fig. 4(g) are primarily from the uncertainties of the measurement of *L*_*ij*_(See Section VII of SI). The differences between theoretical and experimental results for I_11_-I_12_, I_11_-I_13_, I_21_-I_22_, and I_31_-I_33_ are 0.15 dB, −0.23 dB, −0.28 dB, and 0.15 dB, respectively, evidencing the validity of our theory that does not take into account the inter-modal coupling of the excitation. However, the differences for I_21_-I_23_ and I_31_-I_32_ are significantly larger, which are 1.19 dB and 1.22 dB, respectively. The reason for the larger differences is that in our fiber, LP_11a_ and LP_11b_ are degenerate modes and they are strongly coupled. For the case that we launch LP_11a_ and receive LP_11b_, a portion of the optical power is coupled from the LP_11a_ mode to LP_11b_ mode as the excitation light in the LP_11a_ mode is propagating in the forward direction. Because B_33_ = 3B_23_, the measured backscattering in LP_11b_ that contains the backscattering from both the forward-propagating LP_11a_ and LP_11b_ modes is larger than what the theory predicts, i.e., experimental I_23_ is larger than theoretical I_23_, and therefore, theoretical I_21_-I_23_ is larger than experimental I_21_-I_23_. The same argument applies to I_31_-I_32_. Using the same method that we developed here, we tested two additional pieces of few-mode optical fibers: one is a step-index fiber and another is a graded-index fiber. Section IX of SI presents the description of the fiber characteristics and the results of the measurement. The results on these three fibers consistently show that in absence of strong coupling as our theory assumes, experimental and theoretical results match, leading us to conclude that our theory on Rayleigh scattering in few-mode optical fiber is valid.

### Inter-modal crosstalk due to Rayleigh forward scattering

For the forward-scattering case, following almost identical derivations as in Section I of SI, we can similarly write down the power of Rayleigh scattering excited by mode *i*, and scattered forward into mode *j* received at *z* = *l*_F_ as:


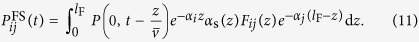


We simplify Eq. (10) by similarly assuming that *α*_s_(*z*) and *F*_*ij*_(*z*), the overall capture fraction for forward-scattering, are independent with *z*, i.e., *α*_s_(*z*) = *α*_s_ and *F*_*ij*_(*z*) = *F*_*ij*_. Additionally, we assume that the incident light in mode *i* is continuous wave (CW) with a constant power *P*_*i*_. Thus,


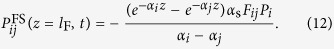


From symmetry consideration, *F*_*ij*_ = *B*_*ij*_, i.e., the corresponding overall capture fraction of forward and backward scattering are identical. Therefore, the inter-modal crosstalk due to Rayleigh forward scattering is


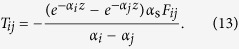


**Figure 5:**
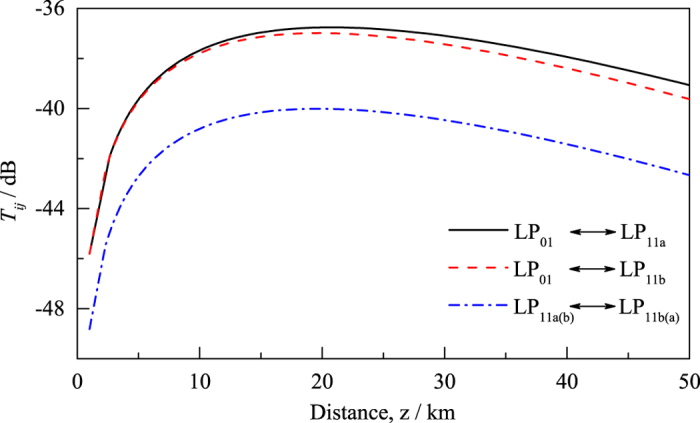
Crosstalk due to inter-modal Rayleigh forward-scattering, as a function of distance. The crosstalk in all the cases studied follows the same pattern: it increases with distance first and then decreases.

Figure 5 is the semi-log plot of the non-diagonal elements of crosstalk due to Rayleigh forward-scattering, as functions of distance. Inter-modal Rayleigh forward-scattering between the LP_01_ and LP_11a(b)_ modes are stronger than that between the LP_11a(b)_ and LP_11b(a)_ modes. Inter-modal Rayleigh forward-scattering increases with *z* at the beginning because the scattered power from mode *i* keeps accumulating in mode *j*. At longer propagation distance, the coupled power decreases as *z* increases because the incident light in mode *i* attenuates due to propagating loss and the scattered light in mode *j* decreases in proportion to the local excitation. A maximum appears in between. Eq. (11) shows that Rayleigh forward-scattering is maximum at


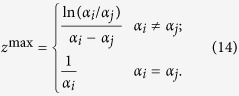


## Discussion

The inter-modal crosstalk induced by forward Rayleigh scattering is difficult to observe experimentally because it is usually shadowed by random mode coupling induced by perturbations and mode coupling resulted from fiber imperfections. One concrete example of fiber imperfections is the imperfections at the interface between the core and cladding layer, which results in so-called small-angle scattering^27^,^28^. Random mode coupling induced by perturbations and mode coupling due to small-angle scattering have not been included in our theoretical analysis that only focuses on the effect of Rayleigh scattering. In our case, *α*_1_ = 0.209 dB/km, *α*_2_ = 0.210 dB/km, and *α*_3_ = 0.233 dB/km. We can use Eq. (14) to get 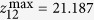 km, 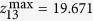 km, and 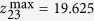 km. The maximum crosstalk due to Rayleigh forward scattering *T*_*ij*_ is - 37 dB, which is 7.51 dB smaller than the minimum total crosstalk (−29.49 dB for the case LP_01_ → LP_11b_) due, dominantly, to random mode-coupling and fiber imperfections that we measured in this three-mode optical fiber (See Section X of SI). Random mode crosstalk can be significantly reduced by, for example, increasing the effective index difference between modes^29^. On the contrary, Rayleigh scattering of optical fibers cannot be significantly reduced because random density fluctuations due to the irregular microscopic structure at the glass softening temperature that are “frozen in”^30^. Hence, inter-modal Rayleigh forward-scattering will likely set the fundamental baseline for crosstalk in MDM systems. This baseline for crosstalk, similar to the baseline for optical attenuation, can be reduced by, for example, using pure-silica core fiber^31^, but cannot be eliminated.

In conclusion, we have systematically studied Rayleigh scattering in few-mode optical fibers. We have established a complete model for both backward and forward scatterings that allows us to calculate the time-dependent, mode-resolved Rayleigh scattering power and have verified our theory experimentally. We have found that Rayleigh forward-scattering is a fundamental mechanism for inter-modal crosstalk. Our theoretical and experimental results show that this crosstalk is 7.51 dB smaller than the total crosstalk measured in the three-mode optical fiber used in the current experiment that is due dominantly to random mode coupling and fiber imperfections. While random mode coupling can be significantly reduced by increasing the effective index difference between modes, crosstalk induced by Rayleigh forward-scattering cannot be reduced much further. We anticipate the impact of our work as follows. First, it can guide the design and optimization of hardware and algorithms of SDM communication systems. Second, it can serve as the foundations for optical time-domain reflectometry that uses Rayleigh scattering to probe the perturbation-induced mode-coupling dynamics in SDM systems. In particular, the theory developed here can further include perturbations to quantitatively model the probing signal in this scenario. Finally, in a broader context, it can be useful for designing and implementing various few-mode active and passive devices, including amplifiers, lasers, and sensors, in which Rayleigh scattering imposes a fundamental performance limitation.

## Methods

### Measurement of Rayleigh backscattering

As shown in Fig. 3, a commercial optical time-domain reflectometer (OTDR) (YOKOGAWA AQ7285) with a 50-dB dynamic range was used to launch optical pulses at the central wavelength of 1550 nm, each with a pulse width of *ΔT* = 100 ns and peak power of *P*_0_ = 37 mW, into a 4.75-km-long three-mode optical fiber through a free-space mode multiplexer/de-multiplexer. The refractive indices of the core and cladding of the fiber are 1.4495 and 1.4440, respectively; the diameter of the core is 13.69 μm; the numerical aperture (NA) of the fiber is 0.1259. The single-pass insertion loss between the first circulator and the last coupling lens of the mode multiplexer/de-multiplexer was 5.85 dB, 9.83 dB and 9.22 dB for the LP_01_, LP_11a_, and LP_11b_ channels, respectively. The coupling losses from free space to the fiber were 0.50 dB, 2.51 dB, and 2.89 dB for the LP_01_, LP_11a_, and LP_11b_ channels, respectively. Section VI of SI details the measurement of optical losses. The reflected light and Rayleigh back-scattering in these three modes were de-multiplexed and then sent back to the OTDR through circulators. We used an acousto-optic modulator (Brimrose AMM-55-8-70-1550-2FP) to eliminate strong optical reflection at the facet (see Section VIII of SI for more details). This reflection would otherwise generate a dead zone approximately from 0 to 1.5 km. Figure 4 shows the configuration when the excitation is in the LP_11b_ mode and the signal collected is in LP_01_ mode. We manually reconfigured the fiber connections for other combinations of excitation and collected channels.

## Additional Information

**How to cite this article**: Wang, Z. *et al*. Rayleigh scattering in few-mode optical fibers. *Sci. Rep.* **6**, 35844; doi: 10.1038/srep35844 (2016).

## Supplementary Material

Supplementary Information
