# Supplementary material for: Rayleigh scattering in few-mode optical fibers
Source: Sci Rep. 2016 Oct 24;6:35844. doi: 10.1038/srep35844 (PMC5075780; doi:10.1038/srep35844)
Supplement: Supplementary Information [file srep35844-s1.pdf]

Supplementary Information for
Rayleigh scattering in few-mode optical fibers

Zhen Wang, [1, 2, †] Hao Wu, [1, 2, †] Xiaolong Hu, [1, 2, *]
Ningbo Zhao,[1, 2] Qi Mo,[3] and Guifang Li[4, 1, #]

[1] School of Precision Instrument and Optoelectronic Engineering, Tianjin University, Tianjin 300072, China

[2] Key Laboratory of Optoelectronic Information Science and Technology, Ministry of Education, Tianjin 300072, China

[3] Wuhan Research Institute of Posts and Telecommunications, Wuhan 430074, China

[4] CREOL, The College of Optics & Photonics, University of Central Florida, Orlando, FL 32816, USA

[†] These authors contributed equally to this work.

[*] xiaolonghu@tju.edu.cn

[#] li@ucf.edu

Content

I. Time-dependent power of Rayleigh back-scattering

II. Far fields of LP modes

III. Field radiated by a dipole

IV. Local capture fraction

V. Overall capture fraction

VI. Measurement of optical losses

VII. Uncertainties of the measured intercepts, I_{ij}

VIII. Reduction of the dead zone by using the acousto-optic modulator

IX. Measurement of Rayleigh backscattering on two additional few-mode optical fibers and the comparison with theory

X. Measurement of inter-modal crosstalk

Reference for Supplementary Information

I. Time-dependent power of Rayleigh back-scattering

We derive Eq. (1) using space-time diagrams. Consider light with power $P(z=0,t,t\geq 0)$ launched into forward-propagating mode i of the few-mode fiber at its one end $z=0$. The total length of fiber is denoted as l_{F}. We now calculate the back-scattered power P_{ij}^{BS} in mode j received at $z=0$.

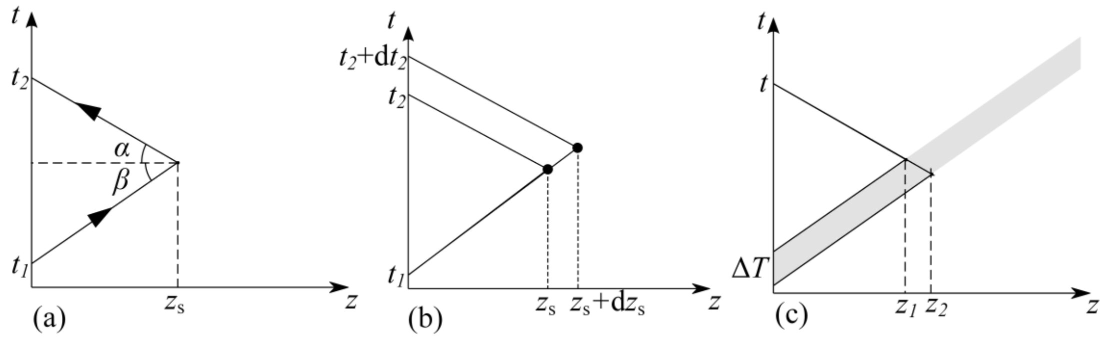

Figure S1 Space-time diagrams for calculating time-dependent power of Rayleigh back-scattering.

We first calculate the differential energy $\mathrm{d}^2E(0,t_2)$, which is the back-scattered energy received at the time t_2 due to the light launched into the fiber at the time interval $[t_1,t_1+\mathrm{d}t_1]$ and scattered by the scatters at the span $[z_{\mathrm{s}},z_{\mathrm{s}}+\mathrm{d}z_s]$. Fig. S1 (a) presents the space-time diagram for this case. The differential energy $\mathrm{d}^2E(0,t_2)$ is

$$\mathrm{d}^2E(0,t_2)=P(0,t_1)\mathrm{d}t_1e^{-\alpha_iz_{\mathrm{s}}}\alpha_{\mathrm{s}}(z_{\mathrm{s}})\mathrm{d}z_{\mathrm{s}}B_{ij}(z_{\mathrm{s}})e^{-\alpha_jz_{\mathrm{s}}}, \tag{S1}$$

where $P(0,t_1)\mathrm{d}t_1$ is the differential energy launched into the fiber; $e^{-\alpha_iz_{\mathrm{s}}}$ takes into account the optical loss of the fiber from $z=0$ to $z=z_{\mathrm{s}}$; $\alpha_{\mathrm{s}}(z_{\mathrm{s}})\mathrm{d}z_{\mathrm{s}}$ is the ratio of total back-scattered energy to the incident energy at $z=z_{\mathrm{s}}$; $B_{ij}(z_{\mathrm{s}})$ is the ratio of the energy coupled into back-propagating mode j to total scattered energy; $e^{-\alpha_jz_{\mathrm{s}}}$ takes into account the optical loss of the fiber from $z=z_{\mathrm{s}}$ to $z=0$ for the back-propagating mode j. Eq. (S1) can be written in the differential form:

$$\frac{\mathrm{d}}{\mathrm{d}t_1}\left[\frac{\mathrm{d}E(0,t_2)}{\mathrm{d}z_{\mathrm{s}}}\right]=P(0,t_1)e^{-(\alpha_i+\alpha_j)z_{\mathrm{s}}}\alpha_{\mathrm{s}}B_{ij}(z_{\mathrm{s}}), \tag{S2}$$

and therefore,

$$\frac{\mathrm{d}E(0,t_2)}{\mathrm{d}z_{\mathrm{s}}}=\int_0^{\infty}P\,(0,t_1)e^{-(\alpha_i+\alpha_j)z_{\mathrm{s}}}\alpha_{\mathrm{s}}B_{ij}(z_{\mathrm{s}})\mathrm{d}t_1. \tag{S3}$$

For P_{ij}^{BS}, we have

$$P_{ij}^{\mathrm{BS}}(t_2) = \frac{\mathrm{d}E(0,t_2)}{\mathrm{d}t_2} = \frac{\mathrm{d}E(0,t_2)}{\mathrm{d}z_{\mathrm{s}}}\frac{\mathrm{d}z_{\mathrm{s}}}{\mathrm{d}t_2}. \tag{S4}$$

In Fig. S1 (a), we have $\cot\alpha = v_{\mathrm{g}i}$, $\cot\beta = v_{\mathrm{g}j}$, and in Fig. S1 (b) we get

$$\mathrm{d}t_2 = \frac{\mathrm{d}z_{\mathrm{s}}}{\cot\alpha} + \frac{\mathrm{d}z_{\mathrm{s}}}{\cot\beta} = \frac{\mathrm{d}z_{\mathrm{s}}}{v_{\mathrm{g}i}} + \frac{\mathrm{d}z_{\mathrm{s}}}{v_{\mathrm{g}j}}, \tag{S5}$$

or, equivalently,

$$\frac{\mathrm{d}z_{\mathrm{s}}}{\mathrm{d}t_2} = \frac{v_{\mathrm{g}i}v_{\mathrm{g}j}}{v_{\mathrm{g}i} + v_{\mathrm{g}j}} \equiv \overline{v}. \tag{S6}$$

In Fig. S1 (a),

$$t_2 - t_1 = \frac{z_{\mathrm{s}}}{\overline{v}}. \tag{S7}$$

From Eqs. (S4), (S6), and (S7), we obtain:

$$P_{ij}^{\mathrm{BS}}(t_2) = \int_0^{\infty} P\,(0, t_2 - \frac{z_{\mathrm{s}}}{\overline{v}}) e^{-(\alpha_i+\alpha_j)z_{\mathrm{s}}} \alpha_{\mathrm{s}}(z_{\mathrm{s}}) B_{ij}(z_{\mathrm{s}}) \mathrm{d}z_{\mathrm{s}}, \tag{S8}$$

which is the general expression for the back-scattered power in mode j received at $z = 0$ due to the excitation in mode i.

Now we consider the specific case in our study.

1. The incident light is an optical pulse with a width of ΔT and a constant power P_0 within ΔT. See Fig. S1 (c).

2. We assume $\alpha_{\mathrm{s}}(z) = \alpha_{\mathrm{s}}$ and $B_{ij}(z) = B_{ij}$, i.e., they are constants along z.

Then,

$$
\begin{aligned}
P_{ij}^{\mathrm{BS}}(t) &= \int_{z_{\mathrm{A}}}^{z_{\mathrm{B}}} P_0\, e^{-(\alpha_i+\alpha_j)z} \alpha_{\mathrm{s}}(z) B_{ij}(z) \mathrm{d}z \\
&= P_0 \alpha_{\mathrm{s}} B_{ij} \int_{z_{\mathrm{A}}}^{z_{\mathrm{B}}} e^{-(\alpha_i+\alpha_j)z}\, \mathrm{d}z \\
&= \frac{P_0 \alpha_{\mathrm{s}} B_{ij}}{-(\alpha_i+\alpha_j)} \left[e^{-(\alpha_i+\alpha_j)z_{\mathrm{B}}} - e^{-(\alpha_i+\alpha_j)z_{\mathrm{A}}} \right],
\end{aligned} \tag{S9}
$$

where $z_{\mathrm{A}} = (t - \Delta T)\bar{v}$ and $z_{\mathrm{B}} = t\bar{v}$. Thus,

$$
\begin{aligned}
P_{ij}^{\mathrm{BS}}(t) &= \frac{P_0 \alpha_{\mathrm{s}} B_{ij}}{-(\alpha_i+\alpha_j)} \left[e^{-(\alpha_i+\alpha_j)t\bar{v}} - e^{-(\alpha_i+\alpha_j)(t-\Delta T)\bar{v}} \right] \\
&= \frac{P_0 \alpha_{\mathrm{s}} B_{ij}}{\alpha_i+\alpha_j} \left[e^{-(\alpha_i+\alpha_j)(t-\Delta T)\bar{v}} - e^{-(\alpha_i+\alpha_j)t\bar{v}} \right].
\end{aligned} \tag{S10}
$$

To further simplify Eq. (S10), we let $\alpha_i + \alpha_j = 2\bar{\alpha}$. Then

$$
\begin{aligned}
P_{ij}^{\mathrm{BS}}(t) &= \frac{P_0 \alpha_{\mathrm{s}} B_{ij}}{-2\bar{\alpha}} \left[e^{-2\bar{\alpha}(t-\Delta T)\bar{v}} - e^{-2\bar{\alpha}t\bar{v}} \right] \\
&= \frac{P_0 \alpha_{\mathrm{s}} B_{ij}}{-2\bar{\alpha}} e^{-2\bar{\alpha}t\bar{v}} \left[e^{2\bar{\alpha}\Delta T\bar{v}} - 1 \right].
\end{aligned} \tag{S11}
$$

If $2\alpha\bar{v}\Delta T \ll 1$, i.e., the pulses are short: $\Delta T \ll \frac{1}{2\alpha\bar{v}}$, (To estimate $\frac{1}{2\bar{\alpha}\bar{v}}$ in our study, we use $\bar{\alpha} = 0.2$ dB/km ≈ 0.04 /km and $\bar{v} \approx 1 \times 10^5$ km/s. Then, $1/(2\bar{\alpha}\bar{v}) = 125$ µs. In our case, $\Delta T = 100$ ns $\ll \frac{1}{2\bar{\alpha}\bar{v}}$.) then $e^{2\bar{\alpha}\bar{v}\Delta T} - 1 = 2\bar{\alpha}\bar{v}\Delta T$,

$$
P_{ij}^{\mathrm{BS}}(t) = P_0 \alpha_{\mathrm{s}} B_{ij} \bar{v} \Delta T e^{-2\bar{\alpha}\bar{v}t}. \tag{S12}
$$

For a single-mode fiber, $i = j, v_{\mathrm{g}i} = v_{\mathrm{g}j}, \alpha_i = \alpha_j = \alpha$, Then

$$
P_{ij}^{\mathrm{BS}}(t) = P_0 \alpha_{\mathrm{s}} B_{ij} \frac{v}{2} \Delta T e^{-2\alpha z} \quad (t = \frac{2z}{v_{\mathrm{g}}}), \tag{S13}
$$

which is the same expression for a single-mode fiber in Ref. [S1] except that we treat α_{s} and B_{ij} as constants along z.

II. Far fields of LP modes

The far fields of LP modes can be calculated using Fraunhofer diffraction formula[S2]:

$$\psi_{\mathrm{F}}(x,y,z)=\frac{e^{jkz}}{j\lambda z}\exp\left[\frac{jk}{2z}(x^2+y^2)\right]\times \int_{-\infty}^{\infty}\int_{-\infty}^{\infty}\psi_{\mathrm{N}}\,(x',y',z=0)\exp\left[-2\pi j(\frac{x}{\lambda z}x'+\frac{y}{\lambda z}y')\right]\mathrm{d}x'\mathrm{d}y'; \qquad \text{(S14)}$$

or, in a spherical coordinate system, for $\theta\approx 0$, we have $z\approx r$ and

$$\psi_{\mathrm{F}}(r,\theta,\phi)=\frac{e^{jkr}}{j\lambda r}\exp\left[\frac{jk}{2r}(r\sin\theta)^2\right]\int_0^{\infty}\int_0^{2\pi}\psi_{\mathrm{N}}\,(R',\phi')\times \exp\left[-2\pi j(\frac{r\sin\theta\cos\theta}{\lambda r}R'a\cos\phi'+\frac{r\sin\theta\sin\theta}{\lambda r}R'a\sin\phi')\right]R'a\mathrm{d}(R'a)\mathrm{d}\phi', \qquad \text{(S15)}$$

where $R'=\frac{R}{a}$. In an optical fiber, $k=k_0n$, $\lambda=\frac{\lambda_0}{n}$, and $n\approx n_1\approx n_2$ for a weak-guiding fiber, then

$$\psi_{\mathrm{F}}(r,\theta,\phi)=\frac{na^2e^{jk_0nr}}{j\lambda_0 r}\exp\left[\frac{jk_0n}{2}(r\sin^2\theta)\right]\times \int_0^{\infty}\int_0^{2\pi}\psi_{\mathrm{N}}\,(R',\phi')\exp[-jk_0naR'\sin\theta(\cos\phi\cos\phi'+\sin\phi\sin\phi')]R'\mathrm{d}R'\mathrm{d}\phi'. \qquad \text{(S16)}$$

For the far field, $\theta\approx 0$, $\exp\left[\frac{jk_0n}{2}r\sin^2\theta\right]\approx 1$, then,

$$\psi_{\mathrm{F}}(r,\theta,\phi)=\frac{na^2e^{jk_0nr}}{j\lambda_0 r}\int_0^{\infty}\int_0^{2\pi}\psi_{\mathrm{N}}\,(r',\phi')\exp[-jk_0naR'\sin\theta\cos(\phi-\phi')]R'\mathrm{d}R'\mathrm{d}\phi', \qquad \text{(S17)}$$

where for LP_{01} mode, the near field is

$$\psi_{\mathrm{N}}=\psi_{\mathrm{N1}}=\begin{cases}\mathrm{J}_0(UR') & R'\le 1\\ \mathrm{J}_0(U)\mathrm{K}_0(WR')/\mathrm{K}_0(W) & R'\ge 1\end{cases}; \qquad \text{(S18)}$$

for $\mathrm{LP}_{11\mathrm{a}}$ mode, the near field is

$$\psi_{\mathrm{N}}=\psi_{\mathrm{N2}}=\begin{cases}\mathrm{J}_1(UR')\cos\phi' & R'\leq 1\\ \mathrm{J}_1(U)\mathrm{K}_1(WR')\cos\phi'/\mathrm{K}_1(W) & R'\geq 1\end{cases}; \quad \text{(S19)}$$

for $\mathrm{LP_{11b}}$ mode, the near field is

$$\psi_{\mathrm{N}}=\psi_{\mathrm{N3}}=\begin{cases}\mathrm{J}_1(UR')\sin\phi' & R'\leq 1\\ \mathrm{J}_1(U)\mathrm{K}_1(WR')\sin\phi'/\mathrm{K}_1(W) & R'\geq 1\end{cases}. \quad \text{(S20)}$$

J_0 and J_1 are zeroth- and first-order Bessel functions of the first kind, respectively; K_0 and K_1 are zeroth- and first-order of modified Bessel functions of the second kind, respectively. U and V are the core and cladding parameters, respectively[S3]. Define $\tilde{\psi}_{\mathrm{N2}}(R')=\psi_{\mathrm{N2}}(R',\phi')/\cos\phi'$, then

$$\psi_{\mathrm{F2}}(r,\theta,\phi)=\frac{na^2e^{jk_0nr}}{j\lambda_0 r}\int_0^{\infty}\tilde{\psi}_{\mathrm{N2}}\,(R')\times \int_0^{2\pi}\cos\phi'\exp[-jk_0naR'\sin\theta\cos(\phi-\phi')]R'\mathrm{d}R'\mathrm{d}\phi'. \quad \text{(S21)}$$

Using the following relations[S4]:

$$e^{j\tilde{x}\cos\tilde{\phi}}=\mathrm{J}_0(\tilde{x})+2\sum_{n=1}^{\infty}j^n\,\mathrm{J}_n(\tilde{x})\cos(n\tilde{\phi}), \quad \text{(S22)}$$

$$\int_0^{2\pi}\cos(m\tilde{\phi})\cos[n(\hat{\tilde{\phi}}-\tilde{\phi})]\mathrm{d}\tilde{\phi}=\begin{cases}0 & m\neq n\\ 2\pi & m=n=0,\\ \pi\cos m\hat{\tilde{\phi}} & m=n\neq 0\end{cases} \quad \text{(S23)}$$

and

$$\mathrm{J}_1(-x)=-\mathrm{J}_1(x), \quad \text{(S24)}$$

we get

$$\int_0^{2\pi} \cos\phi' \exp[jk_0 n a \underbrace{R'}_{\tilde{x}} \sin\theta \cos(\phi \underbrace{-}_{\tilde{\phi}} \phi')] \mathrm{d}\phi'$$

$$= \int_0^{2\pi} \cos\phi' \left\{ \mathrm{J}_0(k_0 n a R' \sin\theta) + 2\sum_{n=1}^{\infty} j^n \mathrm{J}_n(k_0 n a R' \sin\theta) \cos[n(\phi - \phi')] \right\} \mathrm{d}\phi' \quad \text{(S25)}$$

$$= 2\pi j \cos\phi \mathrm{J}_1(k_0 n a R' \sin\theta).$$

Therefore,

$$\psi_{\mathrm{F2}}(r,\theta,\phi) = -\frac{k_0 n a^2}{r} e^{jk_0 n r} \int_0^{\infty} \tilde{\psi}_{\mathrm{N2}}(R',\phi) \mathrm{J}_1(k_0 n a R' \sin\theta) R' \mathrm{d}R'. \quad \text{(S26)}$$

Similarly,

$$\psi_{\mathrm{F3}}(r,\theta,\phi) = -\frac{k_0 n a^2}{r} e^{jk_0 n r} \int_0^{\infty} \tilde{\psi}_{\mathrm{N3}}(R',\phi) \mathrm{J}_1(k_0 n a R' \sin\theta) R' \mathrm{d}R', \quad \text{(S27)}$$

and

$$\psi_{\mathrm{F1}}(r,\theta,\phi) = -\frac{k_0 n a^2}{r} e^{jk_0 n r} \int_0^{\infty} \psi_{\mathrm{N1}}(R',\phi) \mathrm{J}_1(k_0 n a R' \sin\theta) R' \mathrm{d}R'. \quad \text{(S28)}$$

III. Field radiated by a dipole

Consider a scatter, S, located at ($r' = R_s, \phi' = \phi_s, z = 0$) in the cylindrical coordinate system, as shown in Fig. S2. Assuming that S is at the cross-section $z = 0$ doesn't affect our calculation of capture fractions.

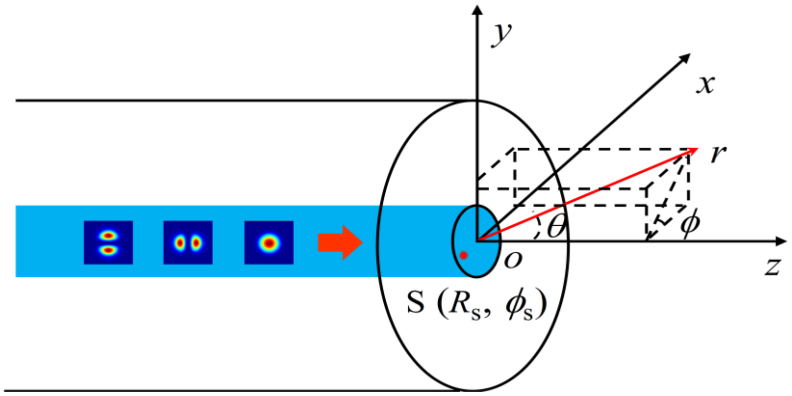

Figure S2 Coordinate systems used for calculating the far fields of LP modes and the field radiated by a dipole.

If S is located at the origin $(r' = 0, \phi' = 0, z = 0)$, then the electric field ψ_s at (r, θ, ϕ) is[S5]

$$\psi_s(r,\theta,\phi) = \frac{1}{4\pi\epsilon_0}\frac{k^2}{r}|\mathbf{P}|\exp(-jkr)\sin\Psi, \tag{S29}$$

where $\mathbf{P} = \kappa\mathbf{E_0}$, κ is the permittivity, a scalar in an isotropic medium, and $\mathbf{E_0}$ is the electric field of incident light. Ψ is the angle between $\mathbf{P}$ and $\mathbf{r}$. We assume that the intensity of the far field $\psi_s(r,\theta,\phi)$ doesn't change if we move the scatter from the origin to $S = (R_s, \phi_s)$; however, the phase of $\psi_s(r,\theta,\phi)$ changes by $\delta(R_s, \phi_s)$:

$$\psi_s(r,\theta,\phi) = \psi_{s0}\frac{1}{r}e^{-j[knr+\delta(R_s,\phi_s)]}\sin\Psi, \tag{S30}$$

where $\psi_{s0} = \frac{\kappa E_0 n^2 k_0^2}{4\pi\epsilon_0}$, R_s has been normalized by the fiber radius, a. If the dipole is polarized along $\mathbf{x}$ direction, then $\cos\Psi = \sin\theta\cos\phi$, and $\sin\Psi = (1-\sin^2\theta\cos^2\phi)^{\frac{1}{2}}$, then

$$\psi_s(r,\theta,\phi) = \psi_{s0}\frac{1}{r}e^{-j[knr+\delta(R_s,\phi_s)]}(1-\sin^2\theta\cos^2\phi)^{\frac{1}{2}}; \tag{S31}$$

if the dipole is polarized along $\mathbf{y}$ direction, then $\cos\Psi = \sin\theta\sin\phi$, and $\sin\Psi = (1-\sin^2\theta\sin^2\phi)^{\frac{1}{2}}$, then

$$\psi_s(r,\theta,\phi) = \psi_{s0}\frac{1}{r}e^{-j[knr+\delta(R_s,\phi_s)]}(1-\sin^2\theta\sin^2\phi)^{\frac{1}{2}}. \tag{S32}$$

Now we calculate $\delta(R_\mathrm{s}, \phi_\mathrm{s})$. Let $\mathbf{r}' = \mathbf{r} - \mathbf{OS}$, then

$$\begin{aligned} r'^2 &= (x - x_\mathrm{s})^2 + (y - y_\mathrm{s})^2 + z^2 \\ &= (x^2 + y^2 + z^2) - 2(xx_\mathrm{s} + yy_\mathrm{s}) + ({x_\mathrm{s}}^2 + {y_\mathrm{s}}^2). \end{aligned} \tag{S33}$$

Using the following relations, $x = r\sin\theta\cos\phi$, $y = r\sin\theta\sin\phi$, $x_\mathrm{s} = aR_\mathrm{s}\cos\phi_\mathrm{s}$, and $y_\mathrm{s} = aR_\mathrm{s}\sin\phi_\mathrm{s}$, we can get

$$r'^2 = r^2 - 2aR_\mathrm{s}r\sin\theta\cos(\phi - \phi_\mathrm{s}) + (aR_\mathrm{s})^2. \tag{S34}$$

Because $r \gg aR_\mathrm{s}$ in the far field, we use $aR_\mathrm{s}\sin\theta\cos(\phi - \phi_\mathrm{s})$ to replace aR_s, then

$$r' \approx r - aR_\mathrm{s}\sin\theta\cos(\phi - \phi_\mathrm{s}). \tag{S35}$$

So, $\delta(R_\mathrm{s}, \phi_\mathrm{s}) = k_0 n(r' - r) \approx -k_0 naR_\mathrm{s}\sin\theta\cos(\phi - \phi_\mathrm{s})$.

In summary, for a dipole located at $(R_\mathrm{s}, \phi_\mathrm{s}, z = 0)$, if it is polarized along **x** direction,

$$\psi_\mathrm{s}(r, \theta, \phi) = \psi_{\mathrm{s}0}\frac{1}{r}e^{-j[knr + \delta(R_\mathrm{s}, \phi_\mathrm{s})]}(1 - \sin^2\theta\cos^2\phi)^{\frac{1}{2}}; \tag{S36}$$

if it is polarized along **y** direction,

$$\psi_\mathrm{s}(r, \theta, \phi) = \psi_{\mathrm{s}0}\frac{1}{r}e^{-j[knr + \delta(R_\mathrm{s}, \phi_\mathrm{s})]}(1 - \sin^2\theta\sin^2\phi)^{\frac{1}{2}}, \tag{S37}$$

where

$$\delta(R_\mathrm{s}, \phi_\mathrm{s}) = k_0 n(r' - r) \approx -k_0 naR_\mathrm{s}\sin\theta\cos(\phi - \phi_\mathrm{s}). \tag{S38}$$

We note that

$$\iint_{2\pi} |\psi_\mathrm{s}|^2 \,\mathrm{d}\Omega = \frac{4\pi}{3r^2}\psi_{\mathrm{s}0}^2. \tag{S39}$$

IV. Local capture fraction

The local capture fraction, b_j, of mode j is defined as the ratio of the back-scattered power coupled into mode j over the total scattered power by the scatter located at $(R_\mathrm{s}, \phi_\mathrm{s})$. b_j can be calculated by the following overlap integral[S1]:

$$b_j(R_\mathrm{s}, \phi_\mathrm{s}) = \frac{1}{2} \frac{\left| \iint_{2\pi} \psi_{\mathrm{F}j} \psi_\mathrm{s} \mathrm{d}\Omega \right|^2}{\iint_{2\pi} \left| \psi_{\mathrm{F}j} \right|^2 \mathrm{d}\Omega \iint_{2\pi} |\psi_\mathrm{s}|^2 \mathrm{d}\Omega}. \tag{S40}$$

where $j = 1,\ 2,\ 3$. For $\mathrm{LP_{11a}}$, we first calculate the numerator of Eq. (S40):

$$\left| \iint_{2\pi} \psi_{\mathrm{F}j} \psi_\mathrm{s} \mathrm{d}\Omega \right|^2 = \frac{k_0^2 n^2 a^4 \psi_{\mathrm{s}0}^2}{r^4} \left| \int_0^{\frac{\pi}{2}} \int_0^{\infty} \tilde{\psi}_{\mathrm{N}2}(R') \mathrm{J}_1(k_0 n a R' \sin\theta) \right| \times R' \mathrm{d}R' \int_0^{2\pi} \cos\phi (1 - \sin^2\theta \cos^2\theta)^{\frac{1}{2}} e^{j k_0 n a R_\mathrm{s} \sin\theta \cos(\phi_\mathrm{s} - \phi)} \mathrm{d}\phi \sin\theta \mathrm{d}\theta. \tag{S41}$$

For $n_1 \approx n_2 \approx n$ in the weakly-guiding few-mode fiber, the far-field distribution only has a significant intensity for small $|\theta|$. Therefore, $\sin\theta \approx \theta$, $1 - \sin^2\theta\cos^2\theta \approx 1$. This approximation means that we have neglected polarizations. The upper limit of the integral over θ in Eq. (S41) can be extended to infinity. Let $\eta = k_0 n a \theta$, then,

$$\left| \iint_{2\pi} \psi_{\mathrm{F}j} \psi_\mathrm{s} \mathrm{d}\Omega \right|^2 = \frac{a^2 \psi_{\mathrm{s}0}^2}{k_0^2 n^2 r^2} \left| \int_0^{\infty} \int_0^{\infty} \tilde{\psi}_{\mathrm{N}2}(R') \mathrm{J}_1(\eta R') R' \mathrm{d}R' 2\pi \cos\phi \mathrm{J}_1(R_\mathrm{s}\eta) \eta \mathrm{d}\eta \right|^2. \tag{S42}$$

Using Hankel transforms[S6],

$$f_2(r_2) = 2\pi \int_0^{\infty} f_1(r_1) \mathrm{J}_m(2\pi r_1 r_2) r_1 \mathrm{d}r_1, \tag{S43}$$

and

$$f_1(r_1) = 2\pi \int_0^{\infty} f_2(r_2) \mathrm{J}_m(2\pi r_2 r_1) r_2 \mathrm{d}r_2, \tag{S44}$$

we obtain

$$\left|\iint_{2\pi}\psi_{\mathrm{F}j}\psi_{\mathrm{s}}\mathrm{d}\Omega\right|^2=\frac{4\pi^2\psi_{\mathrm{s}0}^2}{k_0^2n^2r^4}[\psi_{\mathrm{N2}}(R_{\mathrm{s}},\phi_{\mathrm{s}})]^2. \tag{S45}$$

Now we calculate the denominator:

$$\iint_{2\pi}|\psi_{\mathrm{F2}}|^2\,\mathrm{d}\Omega=\frac{k_0^2n^2a^4}{r^2}\int_0^{\frac{\pi}{2}}\int_0^{2\pi}\cos^2\phi'\mathrm{d}\phi'\times \\ \left|\int_0^{\infty}\tilde{\psi}_{\mathrm{N2}}(R')\mathrm{J}_1(k_0naR'\sin\theta)R'\mathrm{d}R'\right|^2\sin\theta\mathrm{d}\theta. \tag{S46}$$

We use the same approximation as we just used for calculating of the numerator:

$$\begin{aligned}\iint_{2\pi}|\psi_{\mathrm{F2}}|^2\,\mathrm{d}\Omega &= \frac{\pi a^2}{r^2}\int_0^{\infty}\left|\int_0^{\infty}\tilde{\psi}_{\mathrm{N2}}(R')\mathrm{J}_1(R'\eta)R'\mathrm{d}R'\right|^2\eta\mathrm{d}\eta \\ &= \frac{\pi a^2}{r^2}\int_0^{\infty}\left|\tilde{\psi}_{\mathrm{N2}}(R')\right|^2R'\mathrm{d}R'.\end{aligned} \tag{S47}$$

Using Eqs. (S45), (S47) and (S39), we obtain

$$b_2(R_{\mathrm{s}},\phi_{\mathrm{s}})=\frac{3}{2k_0^2n^2a^2}\frac{|\psi_{\mathrm{N2}}|^2}{\int_0^{\infty}\left|\tilde{\psi}_{\mathrm{N2}}(R')\right|^2R'\mathrm{d}R'}. \tag{S48}$$

Similarly,

$$b_3(R_{\mathrm{s}},\phi_{\mathrm{s}})=\frac{3}{2k_0^2n^2a^2}\frac{|\psi_{\mathrm{N3}}|^2}{\int_0^{\infty}\left|\tilde{\psi}_{\mathrm{N3}}(R')\right|^2R'\mathrm{d}R'}, \tag{S49}$$

and

$$b_1(R_{\mathrm{s}},\phi_{\mathrm{s}})=b_1(R_{\mathrm{s}})=\frac{3}{4k_0^2n^2a^2}\frac{|\psi_{\mathrm{N1}}|^2}{\int_0^{\infty}|\psi_{\mathrm{N1}}(R')|^2R'\mathrm{d}R'}. \tag{S50}$$

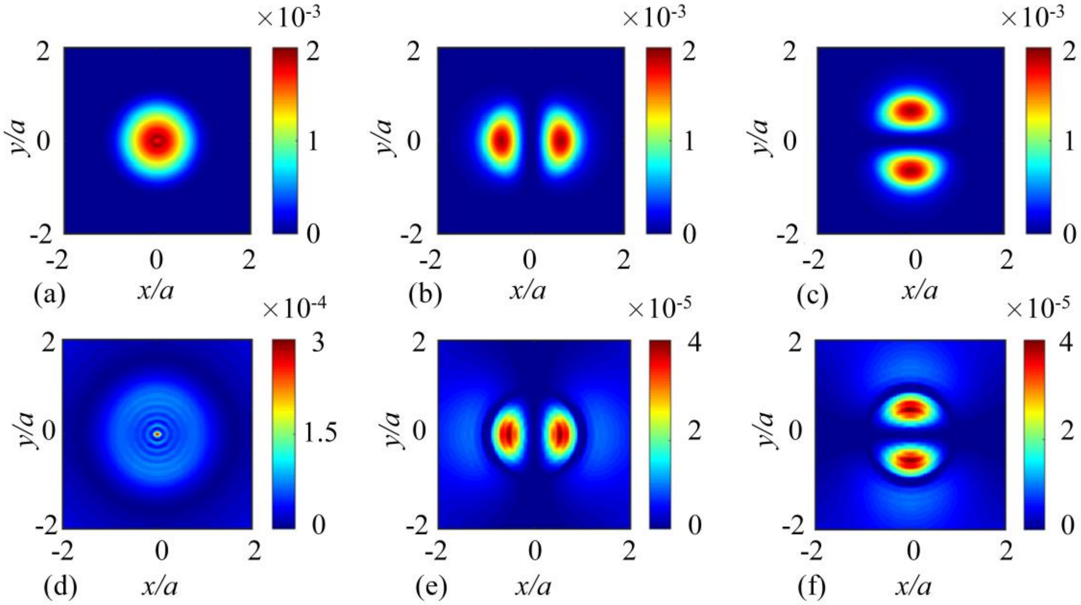

Figure S3 Justification of the approximations used for calculating local caption fractions. (a), (b), and (c) are local caption fractions calculated without using approximations for LP_{01}, LP_{11a}, and LP_{11b} modes, respectively. (d), (e), and (f) are the absolute value of the difference between the calculations with and without using the approximations for LP_{01} , LP_{11a} , and LP_{11b} modes, respectively.

To confirm the validity of the approximation used above, we calculate $\tilde{b}_j$ using Eq. (S40) without approximations. $\tilde{b}_j$ and $|b_j - \tilde{b}_j|$ is presented in Fig. S3. The maximum difference of $|b_j - \tilde{b}_j|$ with and without approximations are 2.62×10^{-4}, 4.27×10^{-5} and 4.27×10^{-5} for LP_{01}, $\mathrm{LP}_{11\mathrm{a}}$ and $\mathrm{LP}_{11\mathrm{b}}$ modes, respectively. The maximum relative difference, $\frac{|b_j - \tilde{b}_j|}{\tilde{b}_j}$, in the core is 6%.

V. Overall capture fraction

The overall capture fraction, B_{ij}, is defined as the back-scattered power coupled into back-propagating mode j over the total scattered power at $z = z_s$. We can explicitly write down $B_{ij} (i = 1,\ 2,\ 3; j = 1,\ 2,\ 3)$ from Eq. (3):

$$\begin{aligned}
B_{11} &= \frac{3}{4k_0^2 n^2 a^2} \frac{\int_0^\infty [\psi_{\mathrm{N1}}(R')]^4 \, R' \mathrm{d}R'}{\left\{\int_0^\infty [\psi_{\mathrm{N1}}(R')]^2 R' \mathrm{d}R'\right\}^2}, \\
B_{12} &= \frac{3}{4k_0^2 n^2 a^2} \frac{\int_0^\infty [\psi_{\mathrm{N1}}(R')]^2 \left[\tilde{\psi}_{\mathrm{N2}}(R')\right]^2 R' \mathrm{d}R'}{\int_0^\infty [\psi_{\mathrm{N1}}(R')]^2 \, R' \mathrm{d}R' \int_0^\infty \left[\tilde{\psi}_{\mathrm{N2}}(R')\right]^2 \, R' \mathrm{d}R'}, \\
B_{13} &= \frac{3}{4k_0^2 n^2 a^2} \frac{\int_0^\infty [\psi_{\mathrm{N1}}(R')]^2 \left[\tilde{\psi}_{\mathrm{N2}}(R')\right]^2 R' \mathrm{d}R'}{\int_0^\infty [\psi_{\mathrm{N1}}(R')]^2 \, R' \mathrm{d}R' \int_0^\infty \left[\tilde{\psi}_{\mathrm{N2}}(R')\right]^2 \, R' \mathrm{d}R'}, \\
B_{21} &= \frac{3}{4k_0^2 n^2 a^2} \frac{\int_0^\infty \left[\tilde{\psi}_{\mathrm{N2}}(R')\right]^2 [\psi_{\mathrm{N1}}(R')]^2 R' \mathrm{d}R'}{\int_0^\infty \left[\tilde{\psi}_{\mathrm{N2}}(R')\right]^2 \, R' \mathrm{d}R' \int_0^\infty [\psi_{\mathrm{N1}}(R')]^2 \, R' \mathrm{d}R'}, \\
B_{22} &= \frac{9}{8k_0^2 n^2 a^2} \frac{\int_0^\infty \left[\tilde{\psi}_{\mathrm{N2}}(R')\right]^4 \, R' \mathrm{d}R'}{\left\{\int_0^\infty \left[\tilde{\psi}_{\mathrm{N2}}(R')\right]^2 R' \mathrm{d}R'\right\}^2}, \\
B_{23} &= \frac{3}{8k_0^2 n^2 a^2} \frac{\int_0^\infty \left[\tilde{\psi}_{\mathrm{N2}}(R')\right]^4 \, R' \mathrm{d}R'}{\left\{\int_0^\infty \left[\tilde{\psi}_{\mathrm{N2}}(R')\right]^2 R' \mathrm{d}R'\right\}^2}, \\
B_{31} &= \frac{3}{4k_0^2 n^2 a^2} \frac{\int_0^\infty \left[\tilde{\psi}_{\mathrm{N2}}(R')\right]^2 [\psi_{\mathrm{N1}}(R')]^2 R' \mathrm{d}R'}{\int_0^\infty \left[\tilde{\psi}_{\mathrm{N2}}(R')\right]^2 \, R' \mathrm{d}R' \int_0^\infty [\psi_{\mathrm{N1}}(R')]^2 \, R' \mathrm{d}R'}, \\
B_{32} &= \frac{3}{8k_0^2 n^2 a^2} \frac{\int_0^\infty \left[\tilde{\psi}_{\mathrm{N2}}(R')\right]^4 \, R' \mathrm{d}R'}{\left\{\int_0^\infty \left[\tilde{\psi}_{\mathrm{N2}}(R')\right]^2 R' \mathrm{d}R'\right\}^2}, \\
B_{33} &= \frac{9}{8k_0^2 n^2 a^2} \frac{\int_0^\infty \left[\tilde{\psi}_{\mathrm{N2}}(R')\right]^4 \, R' \mathrm{d}R'}{\left\{\int_0^\infty \left[\tilde{\psi}_{\mathrm{N2}}(R')\right]^2 R' \mathrm{d}R'\right\}^2}.
\end{aligned} \tag{S51}$$

Eq. (S51) shows the following relations:

$$B_{12} = B_{21} = B_{13} = B_{31}, \tag{S52}$$

and

$$B_{22} = B_{33} = 3B_{23} = 3B_{32}. \tag{S53}$$

VI. Measurement of optical losses

In order to compare the theoretical and experimental results, we carefully measured optical losses. Table S1 lists measured additional losses, L_{ij}, other than the optical attenuation losses in the few-mode fiber. The additional losses includes the insertion losses of two circulators (for laser light), the insertion losses of the spatial mode-multiplexer/de-multiplexer (for both laser light and Rayleigh back-scattering), coupling loss from free space to the few-mode fiber (for laser light), coupling loss from free space to the single-mode fiber (for Rayleigh back-scattering), the insertion loss of the AOM (for Rayleigh back-scattering), and the insertion loss of one (if $i \neq j$) or two (if $i = j$) circulators (for Rayleigh back-scattering). We further corrected the experimental curves by taking into account L_{ij}:

$$\frac{1}{2}(P_{ij}^{\mathrm{BS}}/P_0)[\mathrm{dB}] = -\alpha z + \frac{1}{2}[10\log_{10}(\alpha_s \overline{v} \Delta T) + 10\log_{10} B_{ij} - L_{ij}][\mathrm{dB}] \quad \text{(S54)}$$

The optical loss, α_i, of each mode in the few-mode fiber is obtained by fitting the OTDR data. Each OTDR curve shows a slope of $\overline{\alpha}$ instead of $2\overline{\alpha}$; that is where the factor $\frac{1}{2}$ in Eq. (S62) comes from. We plot $\frac{1}{2}[P_{ij}^{\mathrm{BS}} - P_0][\mathrm{dBm}]$ in Fig. 4 to compare with experimental data.

Table S1 Additional optical losses measured for each combination of excitation and Rayleigh back-scattering

Loss (dB)	$j = \mathrm{LP}_{01}$	$j = \mathrm{LP}_{11a}$	$j = \mathrm{LP}_{11b}$
$i = \mathrm{LP}_{01}$	17.25±0.08	22.25±0.05	21.58±0.08
$i = \mathrm{LP}_{11a}$	22.05±0.08	29.41±0.06	27.56±0.09
$i = \mathrm{LP}_{11b}$	21.83±0.08	28.01±0.05	28.52±0.08

VII. Uncertainties of the measured intercepts, I_{ij}

We analyze the uncertainties of the measured intercepts of the OTDR curves, I_{ij}. The analytical expression of I_{ij} can be obtained by simply setting z = 0 in Eq. (S4). The dominant source of uncertainties is the uncertainties on the measured insertion losses, L_{ij}. Other two terms in Eq. (S4) were measured by OTDR averaging over 30 seconds; their uncertainties are negligibly small compared with the uncertainties of L_{ij}. The least-square fittings of OTDR curves generate errors of I_{ij} on the order of approximately 10^{-4} dB, which can also be neglected. Thus, the uncertainties of the measured intercepts of the OTDR curves, I_{ij}, is $0.5\delta L_{ij}$, where δL_{ij} is the uncertainty of L_{ij}, as we include in Table S1. In Fig. 4 (g) in the main text, the error bar on experimental I_{11}-I_{12}, is therefore calculated by $\pm 0.5\sqrt{(\delta L_{11})^2 + (\delta L_{12})^2}$. The error bars for other cases are similarly calculated.

VIII. Reduction of the dead zone by using the acousto-optic modulator

Figure S4 presents the OTDR data measurement with and without using the acousto-optic modulator (AOM). Without using the AOM, the giant peak due to reflection generates a dead zone approximately from 0 to 1.5 km. Clearly, the AOM reduced the reflection and the dead zone.

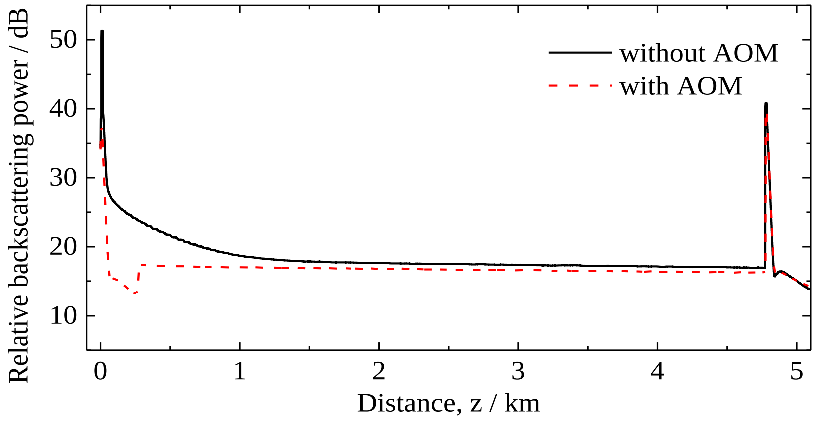

Figure S4 Optical time-domain reflectometry using and without using an acousto-optical modulator (AOM). The AOM reduced the dead zone that was generated by the giant peak due to reflection at the fiber facet.

IX. Measurement of Rayleigh backscattering on two additional few-mode optical fibers and the comparison with theory

Using the same method described in the main text, we measured Rayleigh backscattering on two additional few-mode optical fibers and compared the experimental and theoretical results.

<u>Additional few-mode optical fiber 1</u>:

This sample is a step-index optical fiber with three guiding modes (LP_{01}, LP_{11a}, and LP_{11b}). Its length is 3 km. The radius of its core is 6.8 μm, its numerical aperture is 0.135, and our measurement was done at the normalized frequency of 3.72. Figure S5 presents the experimental and theoretical results.

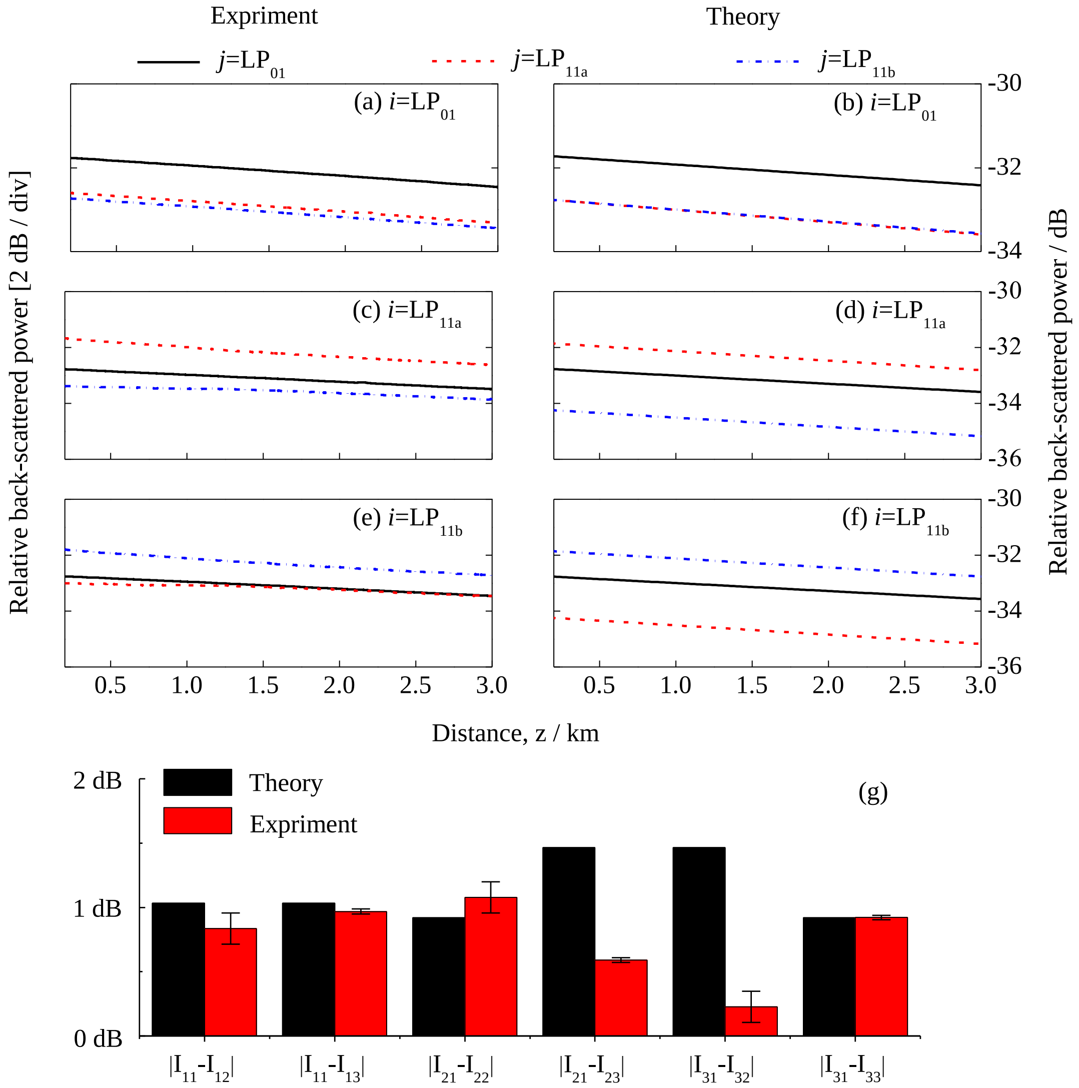

Figure S5 Optical-time-domain-reflectometer measurement of Rayleigh back-scattering in additional few-mode optical fiber 1 and the corresponding theoretical results. (a) experimental results with LP_{01} excitation; (b) theoretical results with LP_{01} excitation; (c) experimental results with LP_{11a} excitation; (d) theoretical results with LP_{11a} excitation; (e) experimental results with LP_{11b} excitation; (f) theoretical results with LP_{11b} excitation; (g) comparison of the experimental and theoretical intercepts relative to I_{i1}.

Additional few-mode optical fiber 2:

This sample is a graded-index optical fiber with three guiding modes (LP_{01}, LP_{11a}, and LP_{11b}). Its length is 10 km. Figure S6 presents the distribution of its refractive index at the wavelength of 1550 nm. For calculating the capture fractions, the modal near fields were simulated by finite-element method using COMOSL Multiphysics. Figure S7 presents the experimental and theoretical results.

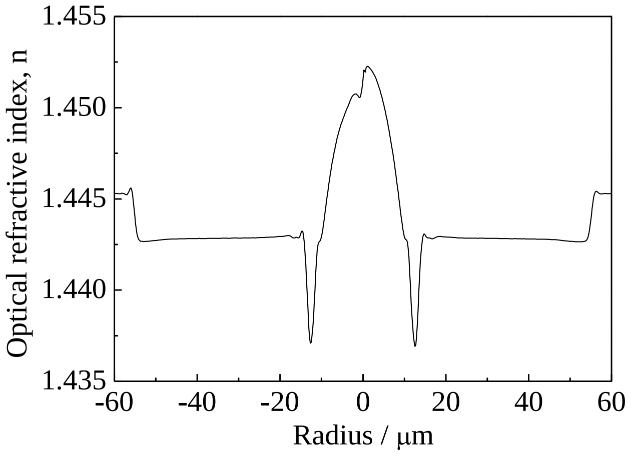

Figure S6 The distribution of its refractive index, at the wavelength of 1550 nm, of additional few-mode optical fiber 2.

Discussion:

Table S2 lists the difference between theoretical and experimental results for the fiber reported in the main text (fiber 0), additional fiber 1, and additional fiber 2. For all three samples, in absence of strong coupling (I_{11}-I_{12}, I_{11}-I_{13}, I_{21}-I_{22}, and I_{31}-I_{33}), the experimental results match the theoretical results, evidencing the validity of our theory; if inter-modal strong coupling exists, as we measured I_{23} and I_{32}, the measured I_{23} and I_{32} are larger than what our theory predicts because the theory does not take into account the inter-modal coupling of the excitation. These analysis has also been presented in detail in the main text.

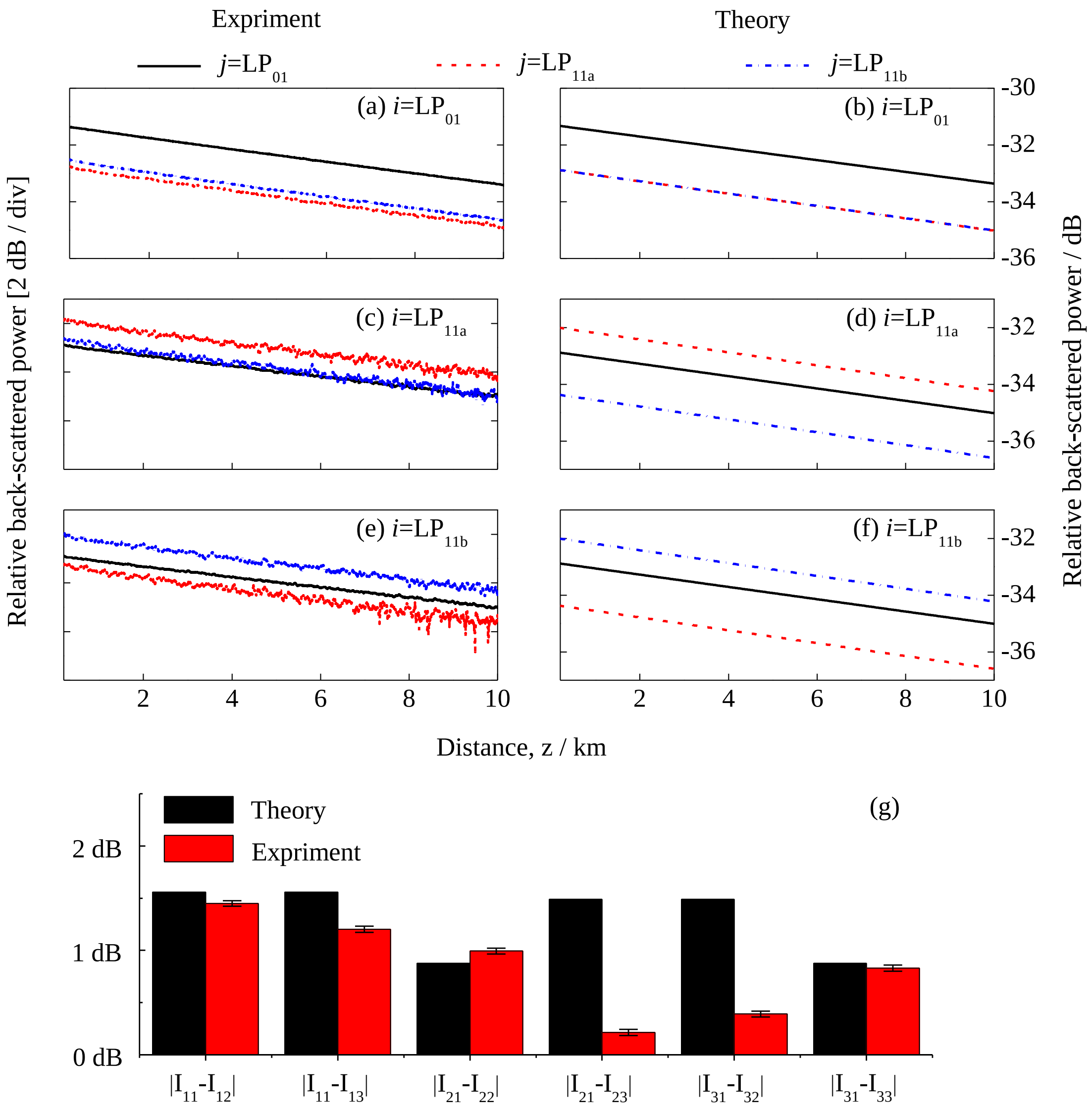

Figure S7 Optical-time-domain-reflectometer measurement of Rayleigh back-scattering in additional few-mode optical fiber 2 and the corresponding theoretical results. (a) experimental results with LP_{01} excitation; (b) theoretical results with LP_{01} excitation; (c) experimental results with LP_{11a} excitation; (d) theoretical results with LP_{11a} excitation; (e) experimental results with LP_{11b} excitation; (f) theoretical results with LP_{11b} excitation; (g) comparison of the experimental and theoretical intercepts relative to I_{i1}.

Table S2 The differences between theoretical and experimental results. The shaded two columns are the cases that inter-modal strong coupling exists in our measurement.

Difference(dB)	$I_{11} - I_{12}$	$I_{11} - I_{13}$	$I_{21} - I_{22}$	$I_{21} - I_{23}$	$I_{31} - I_{32}$	$I_{32} - I_{33}$
Fiber 0	0.15	-0.23	-0.28	1.19	1.22	-0.15
Fiber 1	0.20	0.07	-0.16	0.88	1.24	0
Fiber 2	0.11	0.36	-0.12	1.27	1.10	0.05

X. Measurement of inter-modal crosstalk

We measured the inter-modal crosstalk of the three-mode optical fiber and made comparison with the crosstalk due only to Rayleigh forward scattering obtained from our theory. Using the method in Ref. S7, the mode-coupling ratio from mode *i* to mode *j* where the excitation is in mode *i* can be obtained by

$$\eta_{ij} = \frac{P_{ij}^{BS}}{P_{ii}^{BS}} = 2h_{ij}l_F + \kappa \qquad \text{(S 55)}$$

where h_{ij} is the mode-coupling coefficient, l_F , as defined before, is the length of the fiber, and κ is a constant.

Figure S8 presents the raw data of η_{ij} and the linear fittings to obtain inter-modal crosstalk $10log(h_{ij}l_F)$. We found that the inter-modal crosstalk of $LP_{01} \rightarrow LP_{11a}$, $LP_{11a} \rightarrow LP_{01}$, $LP_{01} \rightarrow LP_{11b}$, and $LP_{11b} \rightarrow LP_{01}$ are -25.34 dB, -21.04 dB, -29.49 dB, and -25.76 dB, respectively. Because the dispersion relations of LP_{11a} and LP_{11b} modes are degenerate, these two modes experience strong coupling. Thus, the analysis of the mode coupling using (S 55) that assumes weak coupling is no longer valid. Indeed, the "net" mode coupling $LP_{11a} \rightarrow LP_{11b}$ obtained using this method was only -33.58 dB; the "net" mode coupling $LP_{11b} \rightarrow LP_{11a}$ was not measurable.

Compared with the inter-modal crosstalk due to Rayleigh forward scattering that is -37 dB at its maximum, as predicted by our theory in the main text, the total crosstalk due to random mode coupling and fiber imperfections is dominantly large.

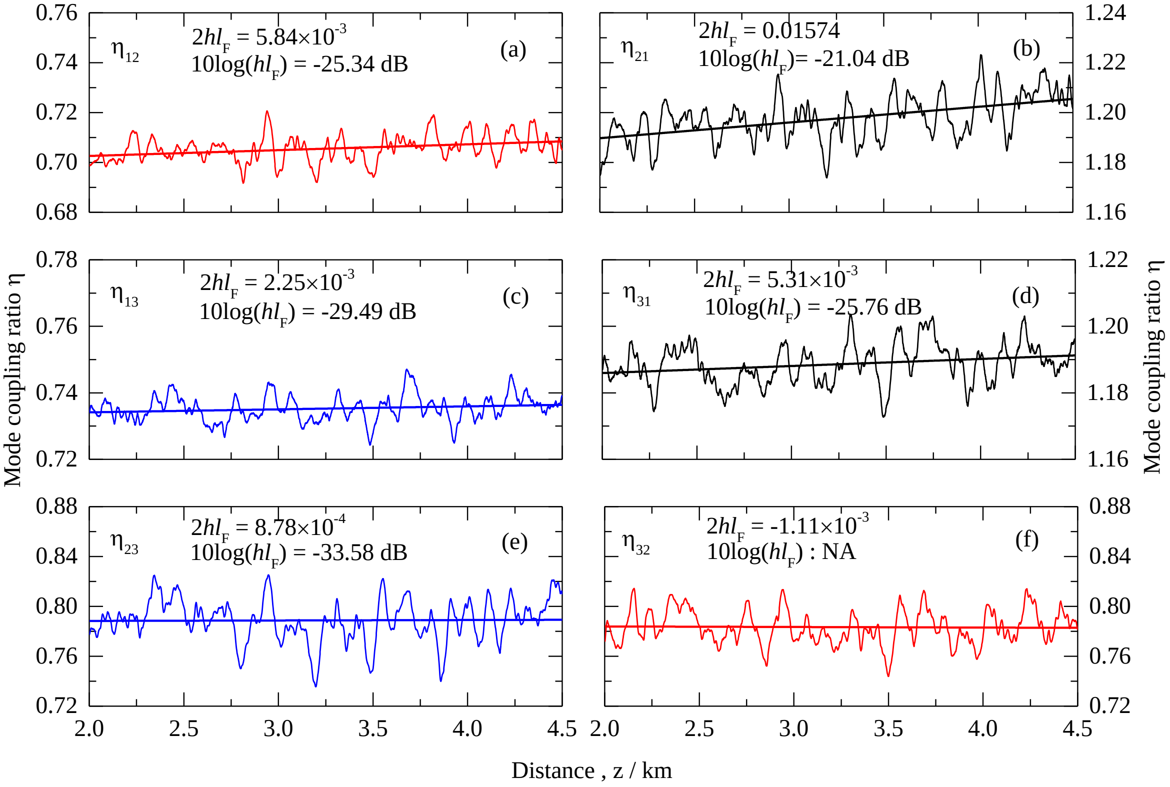

Figure S8 Measure of inter-modal crosstalk of the three-mode optical fiber, using optical time-domain reflectometry. (a), (b), (c), and (d) show the mode coupling of $LP_{01} \rightarrow LP_{11a}$, $LP_{11a} \rightarrow LP_{01}$, $LP_{01} \rightarrow LP_{11b}$,and $LP_{11b} \rightarrow LP_{01}$, respectively. (e) and (f) show the raw data and fittings for $LP_{11a} \rightarrow LP_{11b}$ and $LP_{11b} \rightarrow LP_{11a}$, respectively.

Reference for Supplementary Information

S1. Hartog, A. H. & Gold, M. P. On the theory of back-scattering in single-mode optical fibers. *J. Lightwave Technol.* **2**, 76-82 (1984).

S2. Born, M. & Wolf, E. Principles of Optics. (Cambridge University Press, 1999).

S3. Snyder, A. W. & Love, J. Optical Waveguide Theory. (Springer, 1983).

S4. Riley, K. F., Hobson, M. P. & Bence, S. J. Mathematical Methods for Physics and Engineering. (Cambridge University Press, 2006).

S5. Griffiths, D. J. Introduction to Electrodynamics. (Addison Wesley, 1999).

S6. Debnath, L. & Bhatta, D. Integral Transforms and Their Applications. (CRC press, 2014).

S7. Nakazawa, M., Yoshida, M. & Hirooka, T. Measurement of mode coupling distribution along a few-mode fiber using a synchronous multi-channel OTDR. Opt. Express **22**, 31299-31309 (2014).
